# Low expression of estrogen receptor β in T lymphocytes and high serum levels of anti-estrogen receptor α antibodies impact disease activity in female patients with systemic lupus erythematosus

**DOI:** 10.1186/s13293-016-0057-y

**Published:** 2016-01-12

**Authors:** Angela Maselli, Fabrizio Conti, Cristiano Alessandri, Tania Colasanti, Cristiana Barbati, Marta Vomero, Laura Ciarlo, Mario Patrizio, Francesca Romana Spinelli, Elena Ortona, Guido Valesini, Marina Pierdominici

**Affiliations:** Department of Therapeutic Research and Medicine Evaluation, Istituto Superiore di Sanità, Rome, Italy; Lupus Clinic, Dipartimento di Medicina Interna e Specialità Mediche, Sapienza University, Rome, Italy; Department of Cell Biology and Neurosciences, Istituto Superiore di Sanità, Rome, Italy; San Raffaele Pisana Institute, Rome, Italy

**Keywords:** Estrogen, Estrogen receptor, Anti-ERα antibodies, T lymphocytes, Systemic lupus erythematosus, Gender, Immunity

## Abstract

**Background:**

Current evidence indicates that estrogens, in particular 17β-estradiol (E2), play a crucial role in the gender bias of autoimmune diseases although the underlying molecular mechanisms have not yet been fully elucidated. Immune cells have estrogen receptors (ERs), i.e., ERα and ERβ, that play pro- and anti-inflammatory functions, respectively, and the presence of one estrogen receptor (ER) subtype over the other might change estrogen effects, promoting or dampening inflammation. In this study, we contributed to define the influences of E2 on T cells from female patients with systemic lupus erythematosus (SLE), a representative autoimmune disease characterized by a higher prevalence in women than in men (female/male ratio 9:1). Particularly, our aim was to evaluate whether alterations of ERα and ERβ expression in T cells from female SLE patients may impact lymphocyte sensitivity to E2 and anti-ERα antibody (anti-ERα Ab) stimulation interfering with cell signaling and display a direct clinical effect.

**Methods:**

Sixty-one premenopausal female patients with SLE and 40 age-matched healthy donors were recruited. Patients were divided into two groups based on the SLE Disease Activity Index 2000 (SLEDAI-2K) (i.e., <6 and ≥6). ER expression was evaluated in T lymphocytes by flow cytometry, immunofluorescence, and Western blot analyses. Serum anti-ERα Ab levels were analyzed by enzyme-linked immunosorbent assay (ELISA). ER-dependent signaling pathways were measured by a phosphoprotein detection kit.

**Results:**

Intracellular ERβ expression was significantly lower in T cells from patients with SLEDAI-2K ≥6 as compared with healthy donors and patients with SLEDAI-2K <6 and negatively correlated with disease activity. The expression of intracellular and membrane-associated-ERα was similar in SLE and control T cells. ER-dependent signaling pathways were activated in T cells from SLE patients with SLEDAI-2K ≥6, but not with SLEDAI-2K <6, when both membrane and intracellular ERs were stimulated by co-treatment with E2 and anti-ERα Abs.

**Conclusions:**

Our results demonstrate an altered ER profile in SLE patients, possibly contributing to SLE pathogenesis and interfering with clinical activity, and highlight the potential exploitation of T cell-associated ERβ as a biomarker of disease activity.

**Electronic supplementary material:**

The online version of this article (doi:10.1186/s13293-016-0057-y) contains supplementary material, which is available to authorized users.

## Background

Most of autoimmune diseases are more common in women and plausible mechanisms for this female predominance include hormonal and genetic factors. Women tend to have a different age at onset and different disease activity than men [1, 2]. Systemic lupus erythematosus (SLE) is a multifactorial and highly polymorphic systemic autoimmune disease that predominantly afflicts women of child-bearing age, where the reported female:male ratio is 8–15:1 [3]. It tends to exacerbate during pregnancy and to remit after menopause. The precise etiology of SLE remains elusive; however, it is known that a complex interaction between genetic, environmental, and hormonal factors promotes the immune dysfunction underlying the pathogenesis of the disease [4]. Immunological defects include aberrant function of T cells that provide help to autoreactive B cells and infiltrate target tissue, production of a wide array of autoantibodies by dysregulated B cells, formation of immune complex that, once deposited, cause tissue injury. SLE most commonly affects the skin and kidneys but can manifest anywhere in the body. Studies in animal models and humans suggest that estrogens, in particular 17β-estradiol (E2), play an important role in the female preponderance observed in SLE and in disease activity although the underlying molecular mechanisms have not yet been fully elucidated [5]. Estrogens have a complex role in inflammation [6] and most of their effects are mediated by two specific intracellular receptors, i.e., estrogen receptor (ER)α and ERβ, which function as ligand-activated nuclear transcription factors producing genomic effects [7]. Estrogen receptors are expressed in different cell types including immune cells [7–9] and the presence of one ER subtype over the other might change estrogen effects, promoting or dampening inflammation [6]. Several studies with mouse models of SLE have suggested a prominent pro-inflammatory role for ERα, contributing to disease progression [10–12]. In particular, ERα deficiency in lupus mice attenuates glomerulonephritis and increases survival [10]. Accordingly, treatment of lupus mice with the ERα-selective agonist propyl pyrazole triol leads to an accelerated disease course and a shortened survival time [11]. On the other hand, ERβ appears to have an anti**-**inflammatory and immunosuppressive effect on lupus mice and administration of the ERβ-selective agonist diarylpropionitrile (DPN) leads to a reduction of autoantibody production and an amelioration of albuminuria [11]. The discovery of membrane-associated ERα (mERα) in different cell types, including lymphocytes [9], has greatly expanded the understanding of estrogen action [13]. Membrane ERα rapidly activates different protein kinase cascades influencing downstream transcription factors to produce non-genomic effects; at the same time, it can modulate intracellular ER action through the phosphorylation of intracellular ERs and their co-activators [14]. The identification in SLE patients of serum autoantibodies that react with mERα (anti-ERα Abs) and act as estrogen agonists [15] has made the scenario even more complex, opening a new path for the research in the estrogen-related receptor activity. To gain additional insight into the mechanisms underlying E2 effects in SLE pathogenesis, we evaluated in T lymphocytes from female SLE patients the occurrence of alterations of ER expression and ER-dependent signaling pathways activated by E2 and/or anti-ERα Abs. We also evaluated the potential association between ER expression levels and disease activity to assess the potential role of ERs as biomarkers of disease activity in SLE.

## Methods

### Patients and biologic samples

Sixty-one premenopausal female patients with SLE, diagnosed according to the American College of Rheumatology (ACR) revised criteria [16], were recruited in this study. The age of participants ranged between 25 and 47 years with a median age of 38 years. All of the patients attended the Lupus Clinic, Rheumatology Unit, Sapienza University of Rome. The control group consisted of 40 age-matched healthy females. Informed consent was obtained from each subject, and the Ethical Committee of "Policlinico Umberto I", Rome, Italy approved the study. Disease activity was scored on the basis of the SLE Disease Activity Index 2000 (SLEDAI-2K) [17]. Median SLEDAI-2K score was 2 (range 0–23). The SLE patients were categorized into two groups on the basis of SLEDAI-2K scores, i.e., <6 (*n* = 38) and ≥6 (*n* = 23). No participant was taking hormone replacement therapy or oral contraceptives. Forty-two patients were taking anti-malarials, 43 were taking prednisone at a dosage ranging from 7.5 to 262.5 mg for week and 20 were using immunosuppressive drugs (13 were taking mycophenolate, 3 were taking cyclosporine, 3 were taking azathioprine, and 1 patient was taking methotrexate). Table 1 summarizes clinical and serological features and current treatment of the patient population. Sera were obtained by standard methods and stored at –80 °C until used.

**Table 1:** Clinical and serological features and current therapy of SLE patients

Characteristic	
Height (mean ± standard deviation; cm)	163 ± 6
Weight (mean ± standard deviation; kg)	59 ± 9
Body mass index (mean ± standard deviation)	23 ± 4
Acute cutaneous lupus [n/tot (%)]	28/61 (46)
Subacute cutaneous lupus [n/tot (%)]	11/61 (18)
Discoid lupus [n/tot (%)]	5/61 (8)
Photosensitivity [n/tot (%)]	31/61 (51)
Serositis [n/tot (%)]	12/61 (20)
Neuropsychiatric lupus [n/tot (%)]	2/61 (3)
Arthritis [n/tot (%)]	39/61 (64)
Glomerulonephritis [n/tot (%)]	20/61 (33)
Hematological features [n/tot (%)]	32/61 (52)
Arterial thrombosis [n/tot (%)]	1/61 (2)
Venous thrombosis [n/tot(%)]	1/61 (2)
Pregnancy morbidity [n/tot (%)]	6/61 (10)
Secondary anti-phospholipid syndrome [n/tot (%)]	6/61 (10)
ANA [n/tot (%)]	61/61 (100)
Anti-dsDNA [n/tot (%)]	46/61 (75)
Anti-Sm [n/tot (%)]	20/61 (33)
LA [n/tot (%)]	11/61 (18)
aCL IgG [n/tot (%)]	15/61 (25)
aCL IgM [n/tot(%)]	13/61 (21)
aβ2GPI IgG [n/tot (%)]	9/61 (15)
aβ2GPI IgM [n/tot (%)]	6/61 (10)
Anti-malarials [n/tot (%)]	42/61 (69)
Prednisone [n/tot (%)]	43/61 (70)
Immunosuppressants [n/tot (%)]	20/61 (33)

*ANA* anti-nuclear antibodies, *anti-dsDNA* anti-double stranded DNA antibodies, *anti-Sm* anti-Smith antibodies, *LA* lupus anticoagulant antibodies, *aCL* anti-cardiolipin antibodies, *aβ2GPI* anti-β2 glycoprotein I antibodies

### ELISA

Enzyme-linked immunosorbent assay (ELISA) was developed as previously described [15]. Briefly, polystyrene plates (Maxisorp, Nunc, Roskilde, Denmark) were coated with the antigen (2 μg/well ERα, Sigma, St. Louis, MO) in 0.05 M NaHCO3 buffer, pH 9.5, and incubated overnight at 4 °C. The plates were blocked with 100 μl/well of 3 % milk, for 1 h at 37 °C. Human sera were diluted in PBS-Tween and 1 % milk (1:100 for total IgG), 100 μl per well. Peroxidase-conjugated goat anti-human IgG (Bio-Rad Laboratories, Richmond, CA) were diluted in PBS-Tween containing 1 % milk (1:3000) and incubated for 1 h at room temperature. *O*-phenylenediamine dihydrochloride (Sigma) was used as a substrate, and the optical density was measured at 490 nm (optical density (OD)_490_). Mean + 3 standard deviations of the OD reading of the healthy donors was considered as the cutoff level for positive reactions. All assays were performed in quadruplicate. Data were presented as the mean OD corrected for background (wells without coated antigen).

### Purification of anti-ERα Abs from patients’ sera

Antibody purification was performed as previously described [15]. Briefly, recombinant ERα (50 μg, Sigma) was spotted onto a nitrocellulose filter and incubated with sera from 10 SLE patients that had an OD >0.5 by ELISA. The antibodies were eluted with 100 mM glycine, pH 2.5, immediately neutralized with 1 M Tris-HCl, pH 8, and dialyzed against PBS. Endotoxin contamination of antibodies was determined by the quantitative chromogenic Limulus amebocyte cell lysate assay (QCL-1000; BioWhittaker, Walkersville, MD). Antibodies from a preparation of intravenous immunoglobulin (IVIG) precipitated by saturated ammonium sulfate solution were used as control.

### Isolation of peripheral blood mononuclear cells and cell culture conditions

Peripheral blood mononuclear cells were isolated by Ficoll-Hypaque density gradient centrifugation and separation of untouched T cells was performed using the Pan T Cell isolation Kit II (Miltenyi Biotec, Bergisch-Gladbach, Germany). The purity of recovered cells, assessed by flow cytometer, was ≥97 %. Cells were cultured in RPMI-1640 medium without phenol red (Gibco BRL, Grand Island, NY) supplemented with 10 % charcoal-stripped FBS (Euroclone, Pero, Milan, Italy), 2 mM glutamine (Sigma), and 50 μg/ml gentamycin (Sigma). For phosphoprotein assay, T cells were cultured for 24 h; E2 (10 nM, Sigma) was added at the beginning of cell culture whereas human anti-ERα Abs (50 μg/ml) or equal amount of IVIG were added 30 min before the end of incubation time. T cells were also treated as described above plus 10-fold molar excess of the high-affinity estrogen receptor antagonist ICI 182,780 (Tocris, Ellisville, MO). To selectively stimulate ERβ, purified T lymphocytes were treated for 48 h with the ERβ agonist DPN (10 nM, Sigma). In this regard, for cytokine production analysis, untreated or DPN-treated cells were activated with 25 ng/ml phorbol myristate acetate (PMA, Sigma) and 1 μg/ml ionomycin for the last 16 h of culture (IFN-γ, IL-2, and IL-4 analysis) or with 50 ng/ml PMA (Sigma) and 1 μg/ml ionomycin (Sigma) for the last 4 h of culture (IL-17 analysis). To inhibit cytokine secretion, 10 μg/ml brefeldin A (Sigma) was added to each condition at the beginning of stimulation with PMA plus ionomycin.

### Flow cytometry

Surface and intracellular phenotyping of T cells was performed by flow cytometry as previously described [9, 18]. Allophycocyanin-conjugated anti-CD3, allophycocyanin- or phycoerythrin (PE)-conjugated anti-CD4, peridinin chlorophyll protein-conjugated anti-CD8, fluorescein isothiocyanate (FITC)-conjugated anti-CD95, PE-conjugated anti-CD25, PE-conjugated anti-HLA-DR, FITC-conjugated anti-IFN-γ, PE-conjugated anti-IL-4 (BD Biosciences, San Jose, CA), FITC-conjugated anti-IL-17A (eBioscience, San Diego, CA), anti-ERα (clone C-542, Abcam, Cambridge, UK), and anti-ERβ (clone 1531, Santa Cruz Biotechnology, Santa Cruz, CA) monoclonal (m)Abs were used. Anti-ER Abs were visualized by FITC-conjugated F(ab’)2 fragment secondary Ab (Abcam). Equal amount of mouse IgG isotype controls were run in parallel. To determine the frequency of T cell subsets, total lymphocytes were first gated by forward and side scatter and then additionally gated for CD4 or CD8 molecule expression. Acquisition was performed on a FACSCalibur flow cytometer (BD Biosciences) and 50,000 events per sample were run. Data were analyzed using the Cell Quest Pro software (BD Biosciences).

### Immunofluorescence analysis

Immunofluorescence analysis was performed as previously described [9]. Briefly, purified T lymphocytes were fixed with 4 % formaldehyde and permeabilized with 0.5 % Triton X-100 in PBS. Cells were stained with anti-ERα (clone C542, Abcam) or anti-ERβ mAbs (clone 1531, Santa Cruz Biotechnology) and then incubated with Alexa Fluor 488-coniugated secondary antibody (Molecular Probes, Eugene, OR). Nuclei were counterstained with Hoechst 33342 (Molecular Probes). Fluorescence was analyzed with an Olympus U RFL microscope (Olympus, Hamburg, Germany).

### SDS-PAGE and Western blot

SDS-PAGE and Western blot were performed as previously described [9]. Briefly, purified T cells were lysed in RIPA buffer (100 mM Tris-HCl, pH 8, 150 mM NaCl, 1 % Triton X-100, 1 mM MgCl) in the presence of complete protease inhibitor mixture. Protein content was determined by the Bradford assay (Bio-Rad Laboratories). Lymphocyte lysates (30 μg/ml) were loaded in 10 % SDS-PAGE. Anti-ERα (clone F-10) and anti-ERβ (clone 1531) mAbs (both from Santa Cruz Biotechnology) were used as primary Abs. Peroxidase-conjugated goat anti-mouse IgG was used as secondary Ab (Bio-Rad Laboratories), and the reactions were developed using the SuperSignal West Pico Chemiluminescent Substrate (Pierce, Rockford, IL). To ensure the presence of equal amounts of protein, the membranes were reprobed with a rabbit anti-human glyceraldehyde 3-phosphate dehydrogenase (GAPDH) Ab (Sigma). Quantification of protein expression was performed by densitometry analysis of the autoradiograms (GS-700 Imaging Densitometer, Bio-Rad Laboratories).

### Phosphoprotein assay

A commercially available multiplex bead-based immunoassay kit (Bio-Plex Phosphoprotein Detection Kit, Bio-Rad Laboratories) was used according to the manufacturer’s protocol to detect the phosphorylation of the mitogen-activated protein kinases extracellular signal-regulated kinase (ERK) and C-Jun N-terminal kinase (JNK), protein kinase B (Akt), and nuclear factor kappa B (NF-kB) in lysates from purified T lymphocytes obtained from SLE patients treated as described above. Data were analyzed with Bio-Plex manager software, version 4.1.1 (Bio-Rad Laboratories) and reported as fluorescence intensity (FI). Values with a coefficient of variation >12 % were excluded.

### Statistics

Statistical significance was determined using the Mann-Whitney *U* test. Correlations were evaluated by using Spearman’s rank correlation test. Linear regression analysis was used to display a best fit line to the data. Statistical analyses were performed using GraphPad Prism, version 5.0 software (GraphPad Software, San Diego, CA). All tests were two-sided, and a *p* value <0.05 was considered statistically significant.

## Results

### Intracellular ERβ expression was reduced in peripheral blood T lymphocytes from SLE patients with SLEDAI-2K scores ≥6 and correlated with disease activity

**Fig. 1:**
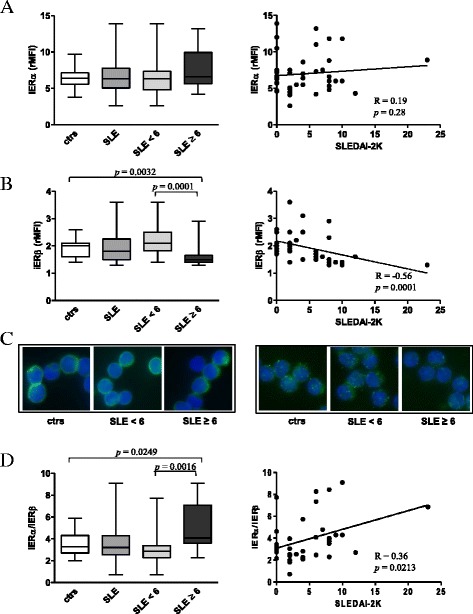
Evaluation of intracellular ER expression levels in T lymphocytes from SLE patients and healthy controls. **a** Intracellular ERα and **b** intracellular ERβ expression levels were evaluated by flow cytometry. Values of ER/isotype control mean fluorescence intensity ratio (*rMFI*) are reported, and data are represented as *box plots* displaying medians, 25th and 75th percentiles as boxes, and the lowest and highest values as whiskers. Statistical differences were calculated by the Mann-Whitney *U* test. Correlations of intracellular ERα and ERβ expression levels with the SLEDAI-2K score are also shown. The Spearman’s rho (*R*) and *p* values were determined using the Spearman’s rank correlation analysis. *Solid lines* represent best fits as estimated by linear regression analysis. **c** Immunofluorescence analysis of intracellular ERα (*left panels*) and ERβ (*right panels*) expression (*green*). Representative images of T lymphocytes from the studied populations are shown. Cell nuclei were stained with Hoechst 33342 in *blue*. Magnification, ×2200. **d** ERα/ERβ ratio and its correlation with the SLEDAI-2K score are shown. Data are represented and analyzed as described above. *ctrs* healthy controls, *iER* intracellular ER, *SLEDAI-2K* Systemic Lupus Erythematosus Disease Activity Index 2000

We first compared the intracellular ERα and ERβ expression in T cells from patients with SLE and healthy controls by flow cytometry and immunofluorescence analyses. Our results indicated that SLE patients showed a greater variability in the expression of ERα (Fig. 1a, left panel) and ERβ (Fig. 1b, left panel) as compared to healthy controls, and no significant differences were detected between these two groups. To estimate whether ER expression level may reflect disease activity, SLE patients were categorized into two groups according to the SLEDAI-2K score at the time of sampling: <6 (inactive/low disease activity) and ≥6 (moderate/high disease activity). No statistically significant differences were detected for ERα expression between SLE T cells from patients with SLEDAI-2K scores ≥6 and those with SLEDAI-2K scores (Fig. 1a, c, left panels).

Additionally, Spearman’s rank analysis did not show any correlation between ERα levels and the SLEDAI-2K score (Fig. 1a, right panel). Differently, a significant lower expression of ERβ was found in T cells from patients with SLEDAI-2K scores ≥6 as compared to those with SLEDAI-2K scores <6 (*p* = 0.0001) and healthy controls (*p* = 0.0032) (Fig. 1b, left panel and Fig. 1c, right panel). Accordingly, a significant negative correlation between ERβ expression in SLE T cells and the SLEDAI-2K score was found (Fig. 1b, right panel, *R* = −0.56; *p* = 0.0001). Western blot analysis of total amount of ERα and ERβ in T cells from SLE patients and healthy controls confirmed the above reported results (Additional file 1: Figure S1). Furthermore, the differential expression of ERβ in SLE T cells and its correlation with disease activity were also observed when CD4^+^ and CD8^+^ T cells were considered separately (Additional file 2: Figure S2). As a result of the minor expression of ERβ in SLE patients with SLEDAI-2K scores ≥6, an increased ERα/ERβ ratio was detected in T lymphocytes from this group of patients as compared to those with SLEDAI-2K scores <6 (*p* = 0.0016) and healthy controls (*p* = 0.0249), Fig. 1d (left panel). ERα/ERβ ratio also positively correlated with the SLEDAI-2K score (Fig. 1d, right panel, *R* = 0.36; *p* = 0.0213). For both ERα and ERβ expression, no further significant associations were found with the epidemiological and clinical parameters evaluated in the present study. In particular, when patients were divided according to the type of medications and dosage schedule (for prednisone, < and ≥7.5 mg/die) at the time of the enrollment, no differences were found for ERα and ERβ expression among the analyzed groups (data not shown).

**Fig. 2:**
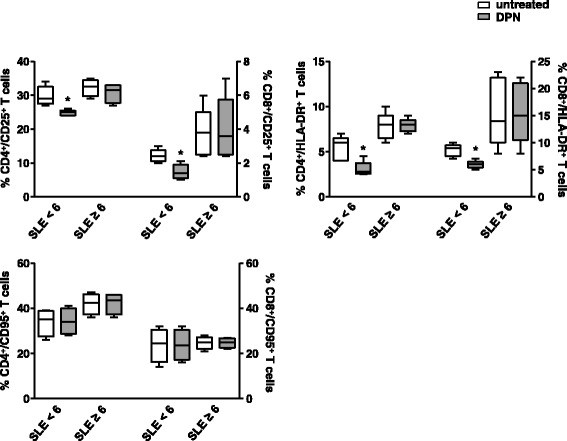
Flow cytometry immunophenotyping of DPN-treated T lymphocytes. Flow cytometry analysis of T cell activation markers was carried out in CD4^+^ and CD8^+^ T lymphocytes from randomly selected SLE patients with SLEDAI-2K <6 and ≥6 (*n* = 5 for group), arbitrarily chosen as representative of the whole series, and treated with DPN (10 nM) for 48 h. Data are represented as *box plots* displaying medians, 25th and 75th percentiles as boxes, and the lowest and highest values as whiskers. *p < 0.05 versus untreated cells

To evaluate whether the differential expression of ERβ in SLE T cells from patients with SLEDAI-2K scores ≥6 and those with SLEDAI-2K scores <6 could affect T cell response to the ERβ-selective agonist DPN, purified T cells from SLE patients were treated with this compound and analyzed for the expression of activation markers, i.e., CD25, HLA-DR, and CD95 molecules, and the production of a panel of cytokines, i.e., IFN-γ, IL-4, and IL-17. As shown in Fig. 2, DPN treatment induced a significant reduction (*p* < 0.05) of CD25 and HLA-DR expression markers both in CD4^+^ and CD8^+^ T cells from SLE patients with SLEDAI 2K scores <6 but not in those with SLEDAI-2K scores ≥6, suggesting that a low expression of ERβ could affect the responsiveness to selective ERβ stimulation. Regarding cytokine production, no significant changes were found in treated versus untreated cells, in both groups of patients (Additional file 3: Figure S3).

### Expression of mERα did not differ in SLE and normal T cells

**Fig. 3:**
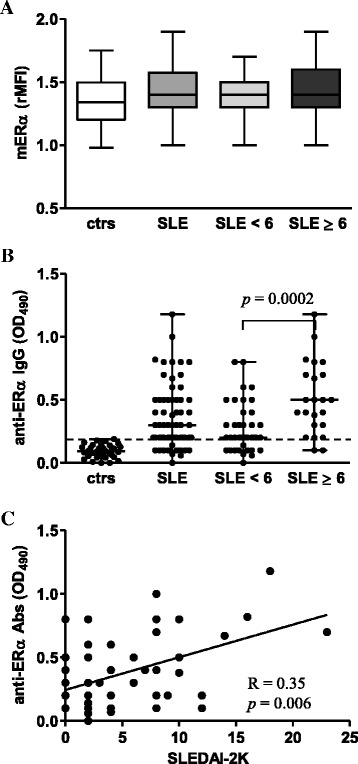
Membrane-associated ERα expression levels and anti-ERα Ab levels. **a** Membrane-associated ERα expression levels were evaluated by flow cytometry. Values of mERα/isotype control mean fluorescence intensity ratio (*rMFI*) are reported, and data are represented as box plots displaying medians, 25th and 75th percentiles as boxes, and the lowest and highest values as whiskers. **b** Anti-ERα Abs (median with range) in sera from patients with SLE and healthy controls. Samples were considered positive if the OD at 490 nm was higher than the cutoff value of an OD at 490 nm of 0.2 (*broken line*). The cutoff value was defined as 3 SD above the mean OD at 490 nm in healthy controls. *Circles* represent individual samples. **c** Correlation of anti-ERα Ab levels with the SLEDAI-2K score. The Spearman’s rho (*R*) and *p* values were determined using the Spearman’s rank correlation analysis. *Solid lines* represent best fits as estimated by linear regression analysis. *ctrs* healthy controls, *mERα* membrane-associated ERα, *OD* optical density, *SLEDAI-2K* Systemic Lupus Erythematosus Disease Activity Index 2000

As a further step, we compared the expression of mERα on T cells from patients with SLE and healthy controls and we found that the level of this receptor was comparable in these two groups (Fig. 3a). When the patients were divided into two groups according to the SLEDAI-2K score (i.e., SLEDAI-2K scores <6 and ≥6), no differences in the expression of mERα between the patients’ subgroups were observed (Fig. 3a). No positivity for mERβ expression was detected in all the studied populations (data not shown). Confirming our previously published results [15], patients with SLE showed detectable serum levels of anti-ERα Abs (Fig. 3b) and a significant difference in the presence of these antibodies between patients with SLEDAI-2K scores <6 and ≥6 was observed (*p* = 0.0002, Fig. 3b). Anti-ERα Ab serum levels significantly correlated with disease activity (*R* = 0.35; *p* = 0.006, Fig. 3c). No correlation was found between mERα expression and anti-ERα Ab serum levels as well as the epidemiological and clinical parameters of the patient population.

### ER-dependent signaling pathways in SLE T cells are influenced by disease activity

**Fig. 4:**
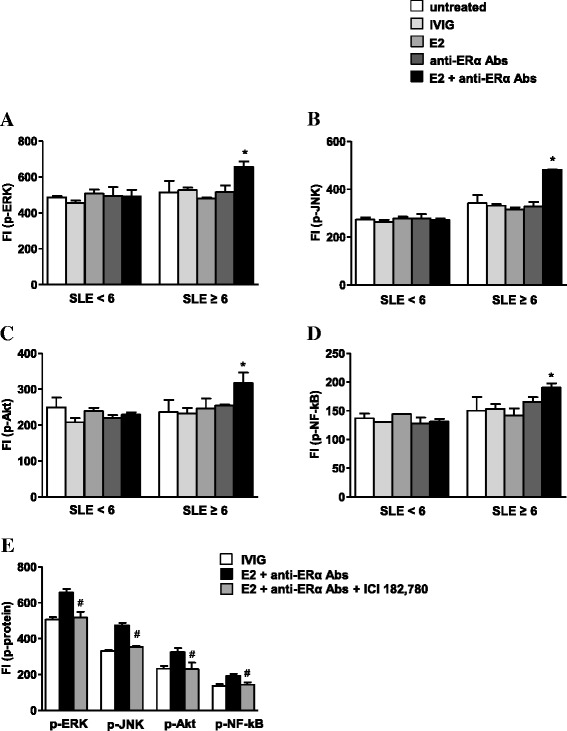
Analysis of ER-dependent signaling pathways in T cells from SLE patients. **a–d** A panel of phosphoproteins was measured using the Bio-Plex Multiplex Phosphoprotein Assay in T cell lysates from randomly selected SLE patients with SLEDAI-2K <6 and ≥6 (*n* = 5 for group), arbitrarily chosen as representative of the whole series, after cell treatment with IVIG, E2, anti-ERα Abs, and E2 plus anti-ERα Abs (see the “Methods” section for details). The fluorescence intensity (*FI*) of **a** phospho (p)-ERK, **b** p-JNK, **c** p-Akt, and **d** p-NF-kB was measured and data are presented as mean ± SD. *p < 0.01 versus untreated cells. **e** Activation of ER with the abovementioned treatments was antagonized by addition of 10-fold molar excess of the high-affinity estrogen receptor antagonist ICI 182,780 in T cells from patients with SLEDAI-2K scores ≥6. Data are presented as mean ± SD. ^#^ *p* < 0.05 versus E2 plus anti-ERα Abs

Based on the above reported data showing a significant negative correlation between ERβ expression and SLEDAI-2K score on the one hand and a significant positive correlation between anti-ERα Ab serum levels and SLEDAI-2K score on the other hand, we compared in T cells from SLE patients with SLEDAI-2K scores <6 and ≥6 the impact of these antibodies and/or E2 on ER-dependent signaling pathways. In particular, we analyzed the activation status of proteins involved in signaling pathways classically targeted by E2 and described as dysregulated in SLE T cells (i.e., the mitogen-activated protein kinases ERK and JNK, Akt, and NF-kB) [19–21]. As shown in Fig. 4a–d, following stimulation with E2 and anti-ERα Abs, ERK, JNK, Akt, and NF-kB activities significantly increased in T cells from patients with SLEDAI-2K scores ≥6 (*p* ≤ 0.01 versus untreated cells), whereas no changes were detectable in those from patients with SLEDAI-2K scores <6. Notably, kinase activity was increased when E2 and anti-ERα Abs were added together but not when the reagents were added alone, suggesting that both membrane and intracellular ER activation are required to observe this effect. To exclude that the modulation of JNK, ERK, Akt, and NF-kB phosphorylation could be due to contaminants in cell culture, we treated SLE T cells with E2 and anti-ERα Abs plus the high-affinity estrogen receptor antagonist ICI 182,780. Addition of this compound to T cells from patients with SLEDAI-2K scores ≥6 significantly reduced (*p* < 0.05) the level of protein phosphorylation in response to ER activation, supporting the specific role of ER in the observed effect (Fig. 4e).

## Discussion

In this study, we characterized the ER profile of T cells from female SLE patients and the susceptibility of these cells to E2 and anti-ERα Ab stimulation, taking into account disease activity. Growing evidence suggests that ER subtypes mediate distinct transcriptional activities when they are co-expressed in the same cells and that the quantity and distribution of these receptors are crucial for their biological effects [6]. Thus, the analysis of ERα and ERβ expression in lymphocytes from SLE patients may provide a useful tool in understanding the responsiveness of these cells to estrogens. In the current study, we observed that the expression of intracellular ERβ was significantly reduced in T cells from SLE patients with SLEDAI-2K scores ≥6 as compared to those with SLEDAI-2K scores <6 and healthy controls. Importantly, a negative correlation between intracellular ERβ expression levels and the SLEDAI-2K score was found. No significant difference was detectable for intracellular and membrane ERα expression between T cells from SLE patients and those from healthy controls. According to what observed by us, Inui et al. [22] found a decreased expression of ERβ mRNA level in peripheral blood mononuclear cells from SLE patients as compared with those from normal controls and a significant inverse correlation between ERβ mRNA expression level and the SLEDAI score. In contrast, Rider et al. [23] did not find any significant difference between SLE and normal T cells in the level of both ERα and ERβ and any association between ER expression and disease activity. In this regard, the discrepancy between our results and those of Rider et al. [23] may be mainly due to the small number of patients enrolled in this latter study. Interestingly, in a recent study by our group, a significant reduction of ERβ expression has been observed in T lymphocytes from patients affected by Crohn disease or ulcerative colitis with active disease as compared to those in remission [24]. In addition, a pro-inflammatory microenviroment has been found to be involved in the downregulation of ERβ [24]. Thus, it is tempting to speculate that also in SLE, the alteration of ERβ expression could be a consequence of the chronic inflammation underlying the active state of the disease. Thus, our results showing a negative correlation between ERβ expression and disease activity open a new path for future research aimed at better defining the role of ERβ in SLE pathogenesis and points to this receptor as a potential marker for disease activity.

In a previous paper, we observed that anti-ERα Abs detectable in sera from SLE patients significantly correlated with disease activity. These autoantibodies act as estrogen agonists, inducing ERK phosphorylation in freshly isolated T cells from healthy controls [15]. In this study, we observed that anti-ERα Abs in the presence of E2, thus mimicking the in vivo physiological conditions of premenopausal women with SLE, were able to activate ER-dependent signaling pathways, i.e., ERK, JNK, Akt, and NF-kB, in T lymphocytes from patient with SLEDAI-2K scores ≥6 but not from those with SLEDAI-2K scores <6. To note, all these signaling pathways are involved in the induction and maintenance of T cell self-tolerance [25–27] and their aberrant expression has been described in several autoimmune diseases including SLE where increased ERK and JNK activities have been found to correlate with disease activity [28]. Our results suggest that the imbalance in the expression of ER subtypes, i.e., the low expression of the anti-inflammatory ERβ and the consequent predominance of ERα signaling, in T cells from female patients with active SLE may impact lymphocyte sensitivity to E2 and to anti-ERα Ab stimulation, interfering with cell signaling and contributing to inflammation and disease activity.

In this paper, we focused on female SLE patients. However, SLE can also occur in males that have a late onset and different clinical features and outcomes, suggesting that distinct male-specific predisposing and/or pathogenetic factors exist [29]. Further studies are required to understand the sex-related aspects of SLE disease susceptibility, clinical features, and outcome, potentially providing new tools for clinical intervention according to sex.

## Conclusions

We provide evidence that ER profile is altered in T cells from female SLE patients and that the extent of ERβ expression reflects disease activity. Additionally, we provide evidence that a different sensitivity of T cells to ER stimulation exists between SLE patients with high versus low disease activity. A deep characterization of factors controlling ER expression and function may increase our knowledge on the pathogenesis of SLE and open new perspectives for the comprehension and management of the disease. Further analysis using ERα- and ERβ-selective agonists will allow to more deeply investigate the ER-mediated responses elicited in chronic inflammatory conditions and to study the potential role of ER as therapeutic target. Moreover, further longitudinal studies are mandatory in order to validate our results and translate them in the clinical practice, assessing the possibility of using ERβ as prognostic marker in female SLE.

## Additional files

Additional file 1: Figure S1. ERα and ERβ Western blot analysis of T-cell lysates from SLE patients and healthy controls. The expression of ERα and ERβ was evaluated in T lymphocytes from SLE patients, divided in patients with SLEDAI-2K scores <6 and ≥6 and healthy controls (*n* = 5 subjects for group). A Data from representative subjects are shown. B Densitometry analysis of protein levels relative to GAPDH is also shown. Values are expressed as mean ± SD. Statistical differences were calculated by the Mann-Whitney *U* test. **p* < 0.001 versus healthy controls and patients with SLEDAI-2K scores <6. Ctrs, healthy controls. (PPT 182 kb) Additional file 2: Figure S2. Flow cytometry analysis of intracellular ERβ expression levels in CD4+ and CD8+ T lymphocytes from SLE patients and healthy controls. Intracellular ERβ expression levels were evaluated by flow cytometry in CD4+ (A, left panel) and CD8+ (B, left panel) T lymphocytes from SLE patients, considered as a whole or divided in patients with SLEDAI-2K scores <6 and ≥6 and healthy controls. Values of ERβ/isotype control mean fluorescence intensity ratio (rMFI) are reported, and data are represented as box plots displaying medians, 25th and 75th percentiles as boxes, and the lowest and highest values as whiskers. Statistical differences were calculated by the Mann-Whitney *U* test. Correlations of intracellular ERβ expression levels in CD4+ (A, right panel) and CD8+ (B, right panel) T lymphocytes from SLE patients with the SLEDAI-2K score are also shown. The Spearman’s rho (*R*) and *p* values were determined using the Spearman’s rank correlation analysis. Solid lines represent best fits as estimated by linear regression analysis. Ctrs, healthy controls; iER, intracellular ER; SLEDAI-2K, Systemic Lupus Erythematosus Disease Activity Index 2000. (PPTX 152 kb) Additional file 3: Figure S3. Flow cytometry immunophenotyping of DPN-treated T lymphocytes. Flow cytometry analysis of cytokine expression at the single cell level was carried out in CD4+ and CD8+ T lymphocytes from randomly selected SLE patients with SLEDAI-2K <6 and ≥6 (*n* = 5 for group), arbitrarily chosen as representative of the whole series, and treated with DPN (10 nM) for 48 h. For CD4+ and CD8+ T lymphocyte subsets, data were expressed as the percentage of each subset within the CD4+ or CD8+ population considered as 100 %. Data are represented as box plots displaying medians, 25th and 75th percentiles as boxes, and the lowest and highest values as whiskers. (PPTX 169 kb)

## Abbreviations

Ab: antibody
Akt: protein kinase B
Ctrs: healthy controls
DPN: diarylpropionitrile
E2: 17-β estradiol
ER: estrogen receptor
ERK: extracellular signal-regulated kinase
FI: fluorescence intensity
FITC: fluorescein isothiocyanate
GAPDH: glyceraldehyde 3-phosphate dehydrogenase
iER: intracellular ER
IVIG: intravenous immunoglobulin
JNK: C-Jun N-terminal kinase
mER: plasma membrane-associated ER
NF-kB: nuclear factor kappa B
OD: optical density
PE: phycoerythrin
PMA: phorbol myristate acetate
rMFI: mean fluorescence intensity ratio
SLE: systemic lupus erythematosus
SLEDAI-2K: SLE Disease Activity Index 2000
