# Supplementary material for: Low expression of estrogen receptor β in T lymphocytes and high serum levels of anti-estrogen receptor α antibodies impact disease activity in female patients with systemic lupus erythematosus
Source: Biol Sex Differ. 2016 Jan 12;7:3. doi: 10.1186/s13293-016-0057-y (PMC4709986; doi:10.1186/s13293-016-0057-y)
Supplement: Additional file 1: Figure S1. — ERα and ERβ Western blot analysis of T-cell lysates from SLE patients and healthy controls. The expression of ERα and ERβ was evaluated in T lymphocytes from SLE patients, divided in patients with SLEDAI-2K scores <6 and ≥6 and healthy controls (n = 5 subjects for group). A Data from representative subjects are shown. B Densitometry analysis of protein levels relative to GAPDH is also shown. Values are expressed as mean ± SD. Statistical differences were calculated by the Mann-Whitney U test. *p < 0.001 versus healthy controls and patients with SLEDAI-2K scores <6. Ctrs, healthy controls. (PPT 182 kb) [file 13293_2016_57_MOESM1_ESM.ppt]

## Slide 1
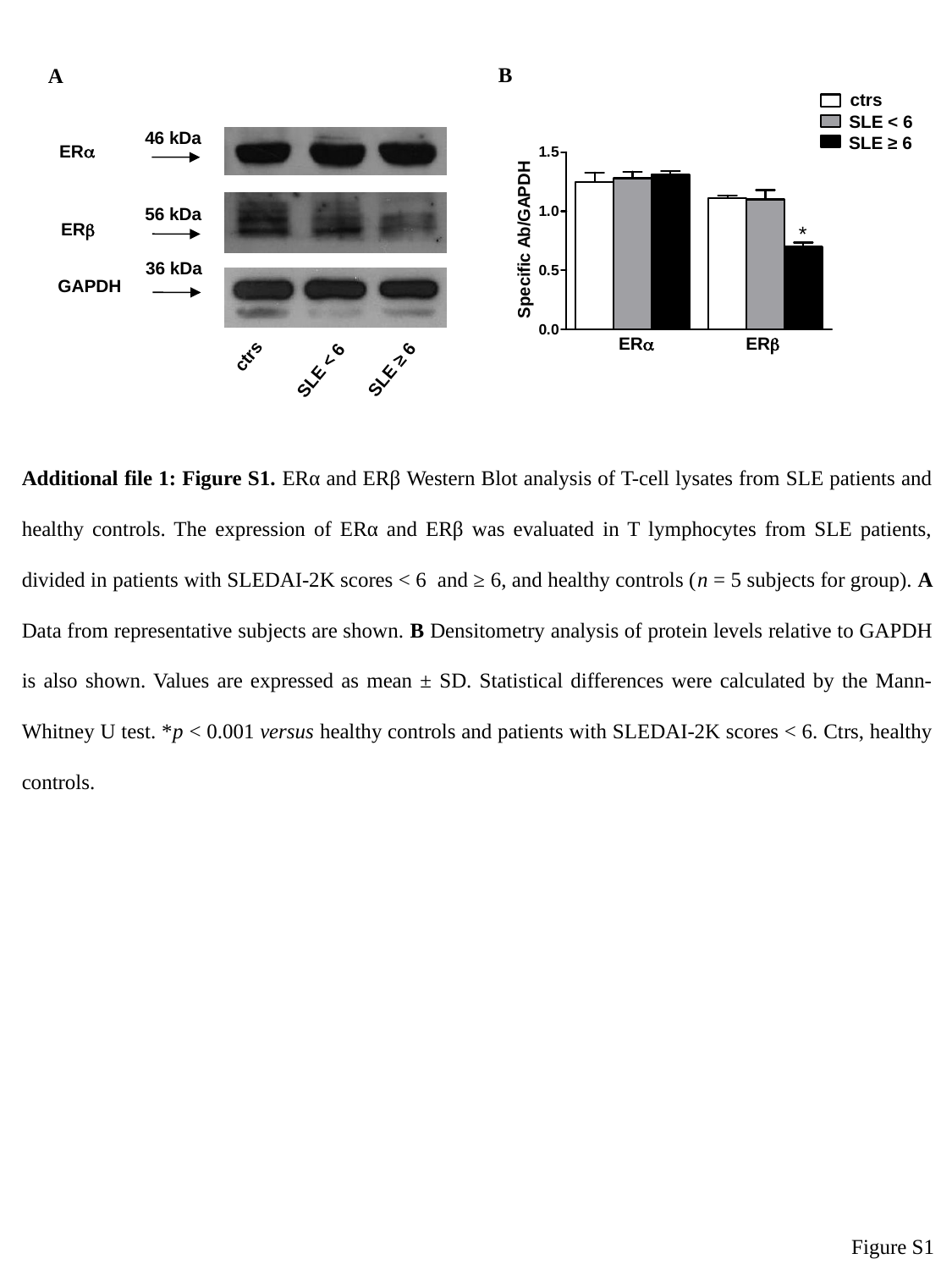

B
A
ctrs
SLE < 6
SLE ≥ 6
*
ERα
ERβ
46 kDa
ERα
56 kDa
ERβ
36 kDa
GAPDH
ctrs
SLE ≥ 6
SLE < 6
Additional file 1: Figure S1. ERα and ERβ Western Blot analysis of T-cell lysates from SLE patients and healthy controls. The expression of ERα and ERβ was evaluated in T lymphocytes from SLE patients, divided in patients with SLEDAI-2K scores < 6 and ≥ 6, and healthy controls (n = 5 subjects for group). A Data from representative subjects are shown. B Densitometry analysis of protein levels relative to GAPDH is also shown. Values are expressed as mean ± SD. Statistical differences were calculated by the Mann-Whitney U test. *p < 0.001 versus healthy controls and patients with SLEDAI-2K scores < 6. Ctrs, healthy controls.
Figure S1
