# Supplementary material for: Low expression of estrogen receptor β in T lymphocytes and high serum levels of anti-estrogen receptor α antibodies impact disease activity in female patients with systemic lupus erythematosus
Source: Biol Sex Differ. 2016 Jan 12;7:3. doi: 10.1186/s13293-016-0057-y (PMC4709986; doi:10.1186/s13293-016-0057-y)
Supplement: Additional file 2: Figure S2. — Flow cytometry analysis of intracellular ERβ expression levels in CD4+ and CD8+ T lymphocytes from SLE patients and healthy controls. Intracellular ERβ expression levels were evaluated by flow cytometry in CD4+ (A, left panel) and CD8+ (B, left panel) T lymphocytes from SLE patients, considered as a whole or divided in patients with SLEDAI-2K scores <6 and ≥6 and healthy controls. Values of ERβ/isotype control mean fluorescence intensity ratio (rMFI) are reported, and data are represented as box plots displaying medians, 25th and 75th percentiles as boxes, and the lowest and highest values as whiskers. Statistical differences were calculated by the Mann-Whitney U test. Correlations of intracellular ERβ expression levels in CD4+ (A, right panel) and CD8+ (B, right panel) T lymphocytes from SLE patients with the SLEDAI-2K score are also shown. The Spearman’s rho (R) and p values were determined using the Spearman’s rank correlation analysis. Solid lines represent best fits as estimated by linear regression analysis. Ctrs, healthy controls; iER, intracellular ER; SLEDAI-2K, Systemic Lupus Erythematosus Disease Activity Index 2000. (PPTX 152 kb) [file 13293_2016_57_MOESM2_ESM.pptx]

## Slide 1
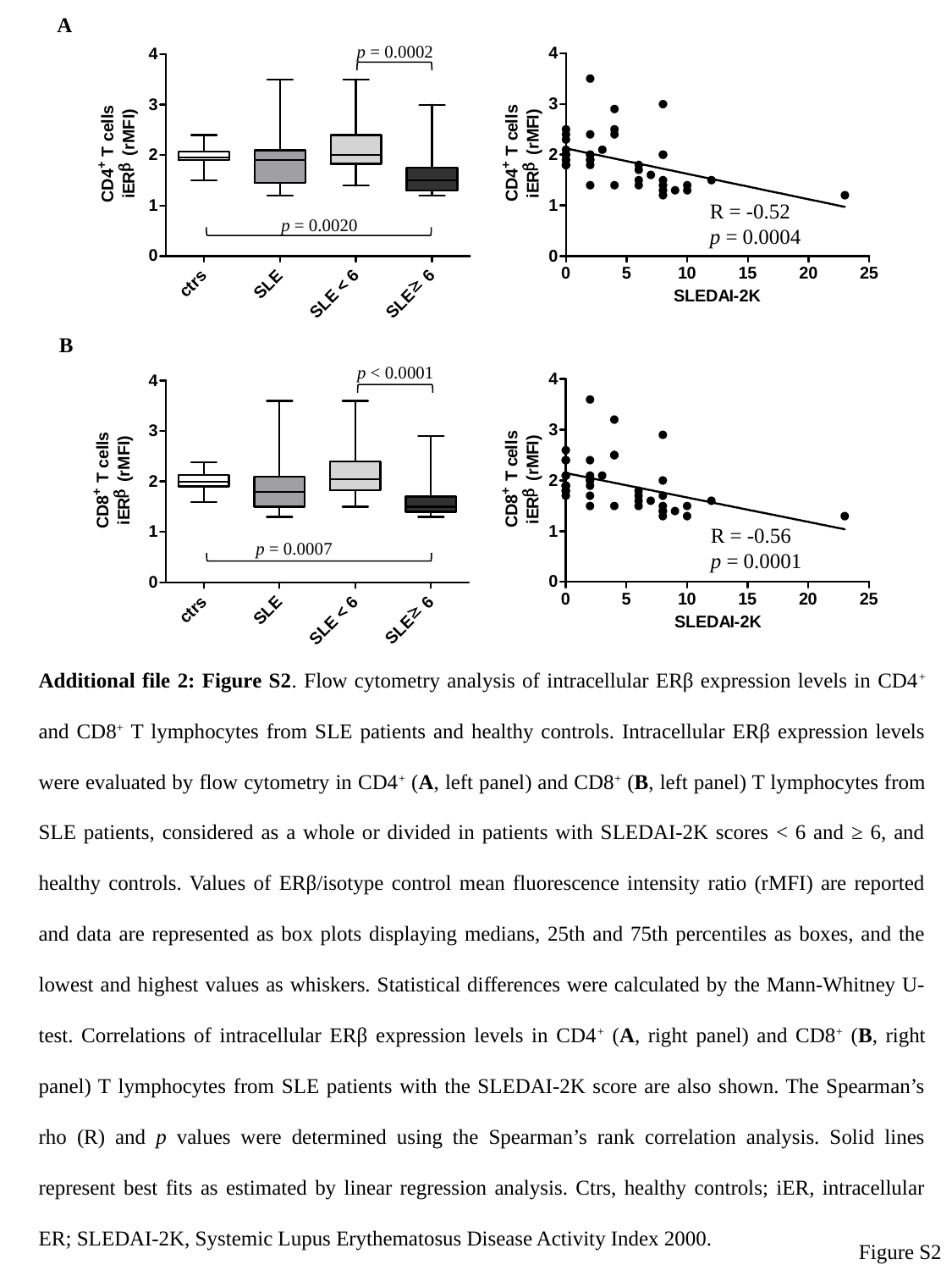

A
p = 0.0002
R = -0.52
p = 0.0004
p = 0.0020
p < 0.0001
R = -0.56
p = 0.0001
p = 0.0007
B
Additional file 2: Figure S2. Flow cytometry analysis of intracellular ERβ expression levels in CD4+ and CD8+ T lymphocytes from SLE patients and healthy controls. Intracellular ERβ expression levels were evaluated by flow cytometry in CD4+ (A, left panel) and CD8+ (B, left panel) T lymphocytes from SLE patients, considered as a whole or divided in patients with SLEDAI-2K scores < 6 and ≥ 6, and healthy controls. Values of ERβ/isotype control mean fluorescence intensity ratio (rMFI) are reported and data are represented as box plots displaying medians, 25th and 75th percentiles as boxes, and the lowest and highest values as whiskers. Statistical differences were calculated by the Mann-Whitney U-test. Correlations of intracellular ERβ expression levels in CD4+ (A, right panel) and CD8+ (B, right panel) T lymphocytes from SLE patients with the SLEDAI-2K score are also shown. The Spearman’s rho (R) and p values were determined using the Spearman’s rank correlation analysis. Solid lines represent best fits as estimated by linear regression analysis. Ctrs, healthy controls; iER, intracellular ER; SLEDAI-2K, Systemic Lupus Erythematosus Disease Activity Index 2000.
Figure S2
