# Supplementary material for: Low expression of estrogen receptor β in T lymphocytes and high serum levels of anti-estrogen receptor α antibodies impact disease activity in female patients with systemic lupus erythematosus
Source: Biol Sex Differ. 2016 Jan 12;7:3. doi: 10.1186/s13293-016-0057-y (PMC4709986; doi:10.1186/s13293-016-0057-y)
Supplement: Additional file 3: Figure S3. — Flow cytometry immunophenotyping of DPN-treated T lymphocytes. Flow cytometry analysis of cytokine expression at the single cell level was carried out in CD4+ and CD8+ T lymphocytes from randomly selected SLE patients with SLEDAI-2K <6 and ≥6 (n = 5 for group), arbitrarily chosen as representative of the whole series, and treated with DPN (10 nM) for 48 h. For CD4+ and CD8+ T lymphocyte subsets, data were expressed as the percentage of each subset within the CD4+ or CD8+ population considered as 100 %. Data are represented as box plots displaying medians, 25th and 75th percentiles as boxes, and the lowest and highest values as whiskers. (PPTX 169 kb) [file 13293_2016_57_MOESM3_ESM.pptx]

## Slide 1
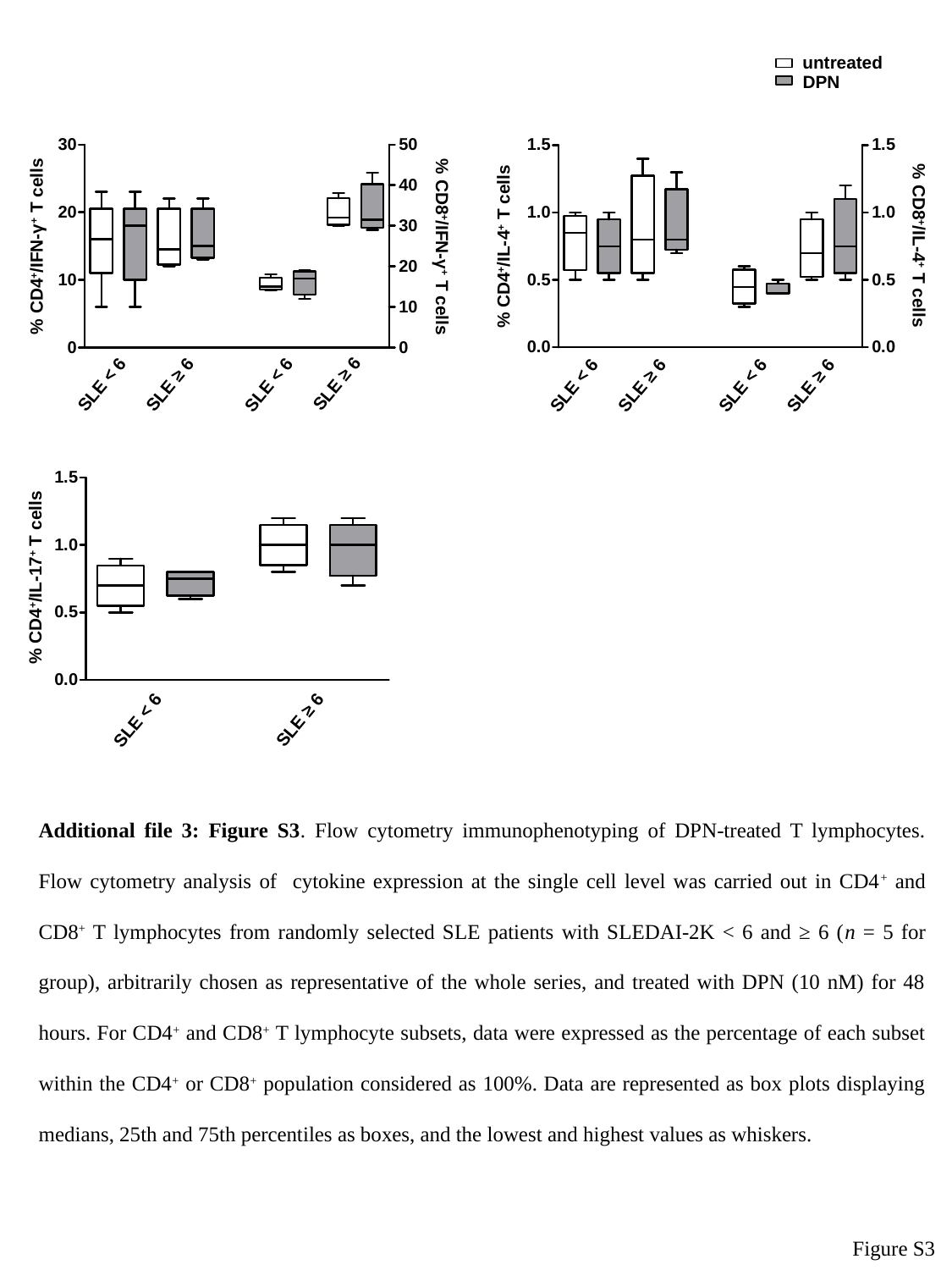

untreated
DPN
% CD4+/IFN-γ+ T cells
% CD8+/IFN-γ+ T cells
SLE ≥ 6
SLE ≥ 6
SLE < 6
SLE < 6
% CD8+/IL-4+ T cells
% CD4+/IL-4+ T cells
SLE ≥ 6
SLE < 6
SLE ≥ 6
SLE < 6
% CD4+/IL-17+ T cells
SLE ≥ 6
SLE < 6
Additional file 3: Figure S3. Flow cytometry immunophenotyping of DPN-treated T lymphocytes. Flow cytometry analysis of cytokine expression at the single cell level was carried out in CD4+ and CD8+ T lymphocytes from randomly selected SLE patients with SLEDAI-2K < 6 and ≥ 6 (n = 5 for group), arbitrarily chosen as representative of the whole series, and treated with DPN (10 nM) for 48 hours. For CD4+ and CD8+ T lymphocyte subsets, data were expressed as the percentage of each subset within the CD4+ or CD8+ population considered as 100%. Data are represented as box plots displaying medians, 25th and 75th percentiles as boxes, and the lowest and highest values as whiskers.
Figure S3
